# Cooperation in binary mixtures of active colloids

**DOI:** 10.1073/pnas.2609894123

**Published:** 2026-10-06

**Authors:** Laura Alvarez, Elena Sesé-Sansa, Demian Levis, Ignacio Pagonabarraga, Lucio Isa

**Affiliations:** ^a^ University of Bordeaux, Centre Nationale de Recherche Scientfique, Centre de Recherche Paul Pascal, UMR 5031, Pessac F-33600, France; ^b^ Laboratory for Soft Materials and Interfaces, This is the unit/division in this affiliation, ETH Zurich, Zurich 8093, Switzerland; ^c^ Editorial Office, American Physical Society, Hauppauge, NY 11788; ^d^ Computing and Understanding Collective Action Lab, Condensed Matter Physics Department, Facultat de Física, Universitat de Barcelona, Barcelona E08028, Spain; ^e^ University of Barcelona Institute of Complex Systems, Barcelona E08028, Spain; ^f^ Condensed Matter Physics Department, Facultat de Física, Universitat de Barcelona, Barcelona E08028, Spain

**Keywords:** active matter, colloids, nonreciprocal, transport, statistical physics

## Abstract

The complex interactions underlying collective motion in biological systems give rise to emergent behaviors such as flocking, sorting, and cooperative transport. These dynamics often involve species with different motilities coordinating movement to optimize navigation and survival. Synthetic analogues based on active colloids offer a controlled platform to explore such behaviors, yet most experimental realizations remain limited to monodisperse systems or mixtures of passive and active particles. Here, we investigate dense binary mixtures of active Janus colloids with two different metal caps leading to distinct motilities and independently tunable mutual alignment, actuated by AC electric fields. We demonstrate experimentally and numerically that binary mixtures form highly dynamic polar clusters. Moreover, particles segregate according to their motility, leading to cooperative “catch-and-run”-like dynamics in which slow particles are locally advected and pushed by fast polar clusters, producing a transient enhancement of slow-particle motility. Our results reveal how motility contrast and alignment combine to drive self-organization in active mixtures, offering strategies for designing reconfigurable materials with collective functionalities.

## Significance

Mixtures of active particles are a minimal setting to study how distinct agents self-organize far from equilibrium. Yet experiments that combine two active species of the same size under identical driving, while keeping interactions tunable, remain rare. We realize such a mixture using AC-field–driven Janus colloids of the same size with matched reorientation dynamics but distinct speeds. Above a density- and alignment-controlled threshold, both species form polar clusters that segregate and exhibit an effective nonreciprocal response: fast clusters advect slow clusters, transiently increasing slow-particle transport. A dry active Brownian particle model with alignment captures and generalizes these emergent cooperative dynamics, providing a minimal framework for designing reconfigurable active materials.

Group motility is ubiquitous in biological systems across various length scales. This dynamical feature arises due to the presence of intricate interactions and of external stimuli, as observed at the microscale in densely populated bacterial (1–3) or amoeba (4) colonies, actin filaments (5, 6), and skin cells (7). Moreover, populations of active biological units with different motilities may exhibit segregation (8, 9), causing spontaneous spatial sorting among species, either via direct competitive mechanisms (10, 11), or by altruistic cooperation of different species to optimize survival (12).

Some of the complex collective dynamics of biological entities is nicely captured by self-propelling (active) colloidal particles, which constitute a model system to single out the essential underlying physics of dense out-of-equilibrium collectives (13–15). These artificial microswimmers convert external sources of energy into net propulsion, leading to emergent dynamical patterns at high enough particle population density. In particular, Janus colloids self-propelling via AC electric fields exhibit rich group behavior due to the complex coupling of hydrodynamic and dipolar interactions, resulting in the formation of particle chains, chiral clusters (16–18), long-range orientational ordered phases (19–21), or very dynamic small polar clusters with short-range orientational order (22, 23).

Mixtures of colloidal populations with distinct motilities have emerged as model systems for uncovering the fundamental physics of multicomponent active matter, motivated by the widespread interactions in nature between entities with different dynamical behaviors. For instance, the addition of active particles to otherwise passive suspensions can strongly remodel collective structure and transport (24–31). Theory and simulations indicate two generic outcomes: active dopants can enhance passive diffusion through persistent forcing (often interpreted as a higher effective temperature), or they can accumulate passives within dense active aggregates and hinder transport (32–36). Mixtures of apolar particles with two motilities have been addressed numerically, mainly in the motility-induced phase separation regime, by varying activity and composition (37). These studies extend kinetic descriptions of active/passive mixtures to active/active systems and motivate analogies to passive mixtures at two effective temperatures (38, 39).

However, experimental realizations of mixed, self-propelled particles are still scarce. Recent work reports demixing for two active colloid populations of different sizes and motility under confinement, based on the emerging nonreciprocity of the two species and their size difference (40). Establishing controlled platforms for active mixtures is therefore crucial to uncover minimal routes by which motility contrast and alignment produce effective nonreciprocal interactions, thereby shaping segregation, cooperation, and competition, and enabling programmable self-organization in reconfigurable active materials (41–44).

In this work, we study the collective behavior of mixed populations of Janus colloids of the same size but with two distinct motilities (fast and slow) at varying densities in the fluid regime under AC electric-field actuation. This setup allows us to disentangle the respective roles of self-propulsion and alignment interactions from other factors, such as particle-size differences, which are known to drive segregation even in the absence of activity (45). We experimentally and numerically identify a confinement-free active–active polar-cluster regime. In this regime, two populations with comparable rotational diffusion but distinct motilities not only segregate, but also interact through repeated fast–slow encounters involving pushing, fragmentation, and transient changes in the motility of both populations. A minimal ABP model with a common symmetric alignment coupling qualitatively reproduces this behavior without imposing explicit microscopic nonreciprocity, showing that an asymmetric cluster-scale response can emerge from motility contrast within a polar phase. At the phenomenological level, the dynamics shares qualitative similarities with the “catch-and-run” behavior reported in mixtures of nonreciprocally coupled conserved scalar fields (31, 46–50). Within this framework, we analyze effective interaction asymmetries between fast and slow populations and discuss when the resulting collective dynamics can be interpreted as effectively nonreciprocal at the coarse-grained level.

## Confinement-Free Polar Clustering of Binary Mixtures

**Fig. 1.**
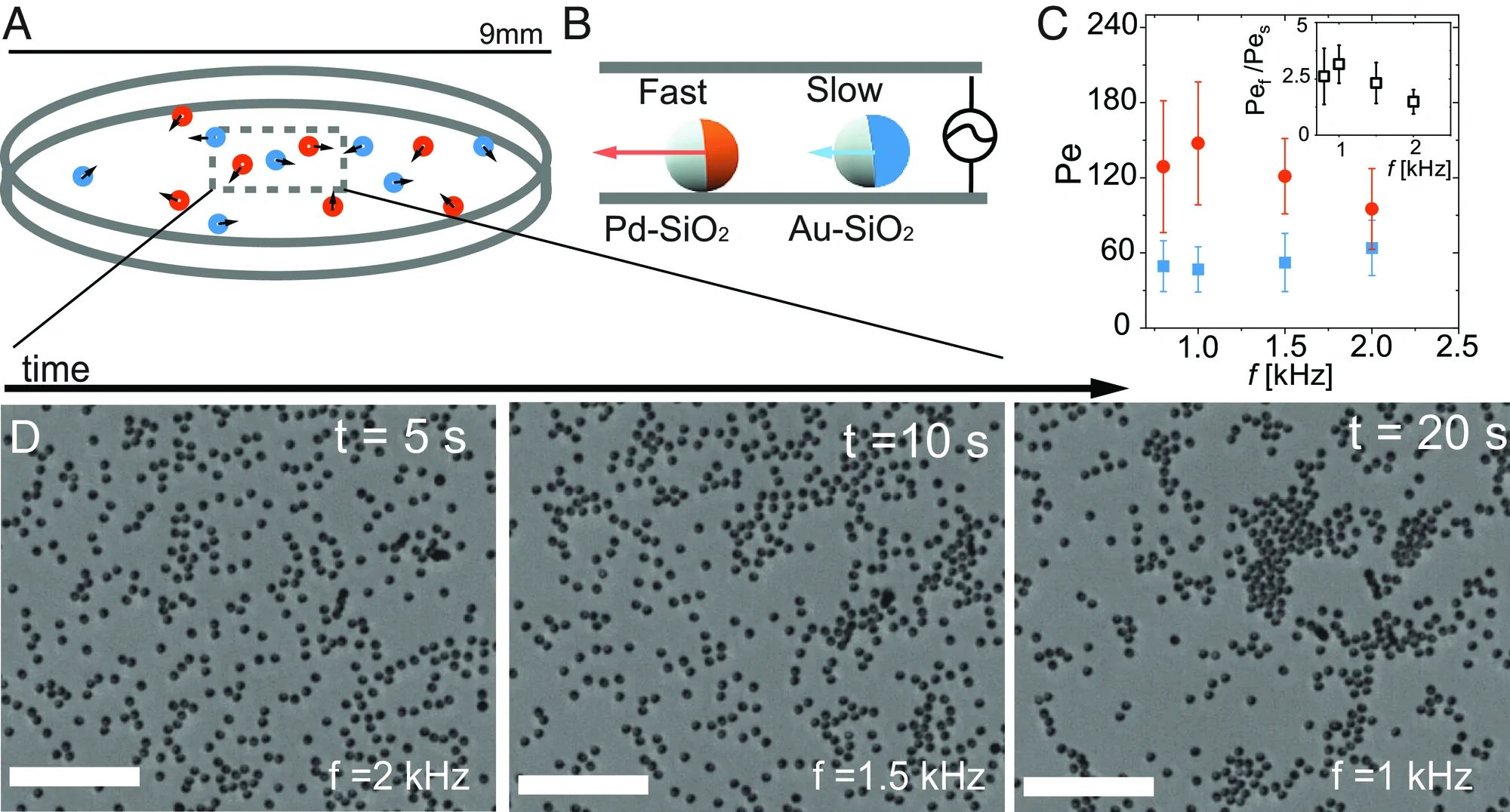
Polar cluster behavior of active colloids. (*A*) Overall schematic representation of the experimental cell (9 mm diameter) with the two different Janus $SiO_{2}$ spheres, i.e., Au-coated ($SiO_{2}$-Au, cyan) and Pd-coated ($SiO_{2}$-Pd, red). (*B*) Illustration of the position of the electrodes on the experimental cell and the application of the transverse AC electric field E. (*C*) Experimental Péclet number $Pe=\frac{v}{\sigma D_{R}}$, as a function of the frequency of the applied field applied for the slow $SiO_{2}$-Au (cyan) and fast $SiO_{2}$-Pd (red). σ=2μm is the particle diameter. The *Inset* represents the ratio of Pe between the fast (Pe_*F*_) and the slow (Pe_*S*_) particles. (*D*) Time sequence bright-field microscopy images of an active mixture showing an overall structure change from disorder to polar cluster phase when changing frequency from 2 kHz (*Left*, disordered), through 1.5 kHz (*Middle*), to 1 kHz (*Right*, polar cluster phase). (Scale bars, 50 μm.).

We design two types of Janus active colloidal species actuated via AC electric field with the same size and reorientation times $\tau_{R}$ but different swimming velocities v. Briefly, we sputter-coat a monolayer of silica ($SiO_{2}$) spheres with a diameter of σ=2μm with i) 2 nm of chromium (Cr) and 6.5 nm of palladium (Pd), and ii) 2 nm of Cr and 4 nm of gold (Au), respectively. The difference in the layer thickness between Au and Pd accounts for the difference in the metal densities ρ ($\rho_{\mathrm{Au}}>\rho_{\mathrm{Pd}}$), to avoid different cap weights for the two-particle species (*SI Appendix*, Fig. S1). The resulting Janus particles are dispersed in MilliQ water and confined between two transparent electrodes consisting of two glass slides coated by a Cr (2 nm) and a Au (10 nm) layer, covered with a thin layer of $SiO_{2}$ (10 nm) to reduce particle sticking, and separated by a 120 μm spacer with an overall area of 254 mm^2^ (Fig. 1 *A* and *B* and *Materials and Methods*). The electrodes are connected to a function generator (Fig. 1*B*) that applies a perpendicular AC electric field between 1 and 10 $V_{\mathrm{pp}}$ (field strength≈8 to 80 kV cm^−1^), and frequencies between 0.8 and 3 kHz. In the kHz regime, both metal-coated Janus particles self-propel close to the bottom electrode, with the $SiO_{2}$ hemisphere in front driven by induced charge electro-osmotic flows (51–54). In this scenario, the unequal polarization of the metal-coated and dielectric hemispheres induces unbalanced fluid flows that lead to particle self-propulsion (53). For each type of Janus particle, we analyze the single-particle dynamics as a function of frequency at a fixed applied voltage amplitude of 8 $V_{\mathrm{pp}}$, starting from dilute conditions at area fraction A %≈ 0.01. We determine the experimental Péclet number $Pe=v/(\sigma D_{R})$ ($D_{R}$ is the rotational diffusivity), as a function of voltage and frequency (Fig. 1*C*) for each particle type (see *SI Appendix*, Fig. S2 for quantification of v and $D_{R}$). We find that the particles exhibit the highest difference in Pe at a voltage of 8 $V_{\mathrm{pp}}$ and a frequency of 1 kHz, with $Pe_{F}^{\exp}$ ($v_{F}=$ 62 ± 17 $\mu m\;s^{-1}$) approximately 2.5 times larger than $Pe_{S}^{\exp}$ ($v_{S}=$ 24 ± 5 $\mu m\;s^{-1}$). Importantly, the measured separation in activity between the two species is significantly larger than the intrinsic sample-to-sample dispersion. In particular, even accounting for the experimental uncertainty and the polydispersity in single-particle motility arising from synthesis variability (e.g., coating defects), the two Péclet-number distributions remain well separated, beyond the overlap expected from the within-species spread. While the $SiO_{2}$ hemisphere is the same in both particles, the difference in swimming velocity arises from the difference in surface conductivity ($\sigma_{S}$) between Pd and Au.

Varying the frequency within our experimental range has a moderate effect on the swimming velocity for both particle types, yet we observe important effect on the particle interactions. In Fig. 1*D* (Movie S1), we illustrate the microstructural change the system exhibits: decreasing the frequency from 2 to 1 kHz, at an area fraction of $\Phi_{A}\approx 0.15$ and fixed voltage $V=8V_{\mathrm{pp}}$, particles aggregate into large-scale clusters. We then characterize the system by the polar order parameter $Z=||1/N\sum_{i}^{N}n_{i}\left|\right|$. At these densities, the system remains disordered with Z=0 at 2 kHz. At 1.5 KHz, the global ordering increases to Z≈0.25, corresponding to the emergence of some local ordering. As the frequency decreases further to f=1 KHz, we observe the emergence of polar clusters with high local order (Z≈1, *SI Appendix*, Fig. S3), and the global alignment parameter averages to Z≈0.6 due to the finite reorientation times and the formation-rupture of the cluster. At these AC field conditions, the clustering can be rationalized as a competition between near-field hydrodynamic coupling and field-induced dipolar interactions (55, 56), which generate an effective aligning torque when particles are at a given threshold distance. In this regime, clusters eventually break due to rotational noise $D_{R}$, a behavior reminiscent of that of dynamical structures found in model systems of self-propelled particles with alignment interactions in a relatively dilute disordered background (57–60).

## Segregation in Nonconfined Binary Mixtures

We further investigated the collective dynamics of dense mixtures of fast $SiO_{2}$–Pd and slow $SiO_{2}$-Au Janus colloids, prepared at an approximate 50:50 population ratio. In view of capturing the main mechanisms responsible for the experimental observations in particle-based simulations, we adopt a simplified “dry” picture, based on the active Brownian particle (ABP) model with alignment interactions (59, 61, 62) (*Materials and Methods*). In this model, the frequency-dependent interactions between colloids are quantified by the dimensionless parameter

[1] $$\begin{array}{c} g=\frac{2J}{D_{R}\pi R_{\varphi }^{2}}; \end{array}$$

which reflects the strength of alignment as compared to rotational noise, and enables the quantitative analysis of its impact on the collective behavior. Here J is the alignment coupling strength, $D_{R}$ the single particle rotational diffusion and $R_{\varphi }$ the alignment interaction range (*Materials and Methods*). This term is a coarse-grained representation of the local polar order generated experimentally by electrohydrodynamic flows and velocity-mediated interactions. Thus, we qualitatively matched the role played by the frequency f in the experiments by varying $g^{-1}$ in the ABP simulations, to recover the polar cluster phase, with Z≈0.5 to 0.7 at $\Phi_{A}\approx 0.2$ over a range of $g^{-1}$ values and Péclet numbers (Pe^sim^ = 3.3, 16.7, 33.3). Although the different metal caps necessarily produce distinct propulsion speeds and electrohydrodynamic flow fields, their polar-ordering behavior remains sufficiently similar (as supported by the phase diagram and polar order parameter Z in *SI Appendix*, Fig. S4) to justify using a common effective g as a minimal approximation. Our simulations show qualitative agreement with experiments, confirming that the onset of polar clusters is primarily governed by particle density and alignment strength ($g^{-1}\approx 0.2$), with minimal influence from the Péclet number (Movie S2), in agreement with mean-field predictions and particle-based simulations (59, 63).

**Fig. 2.**
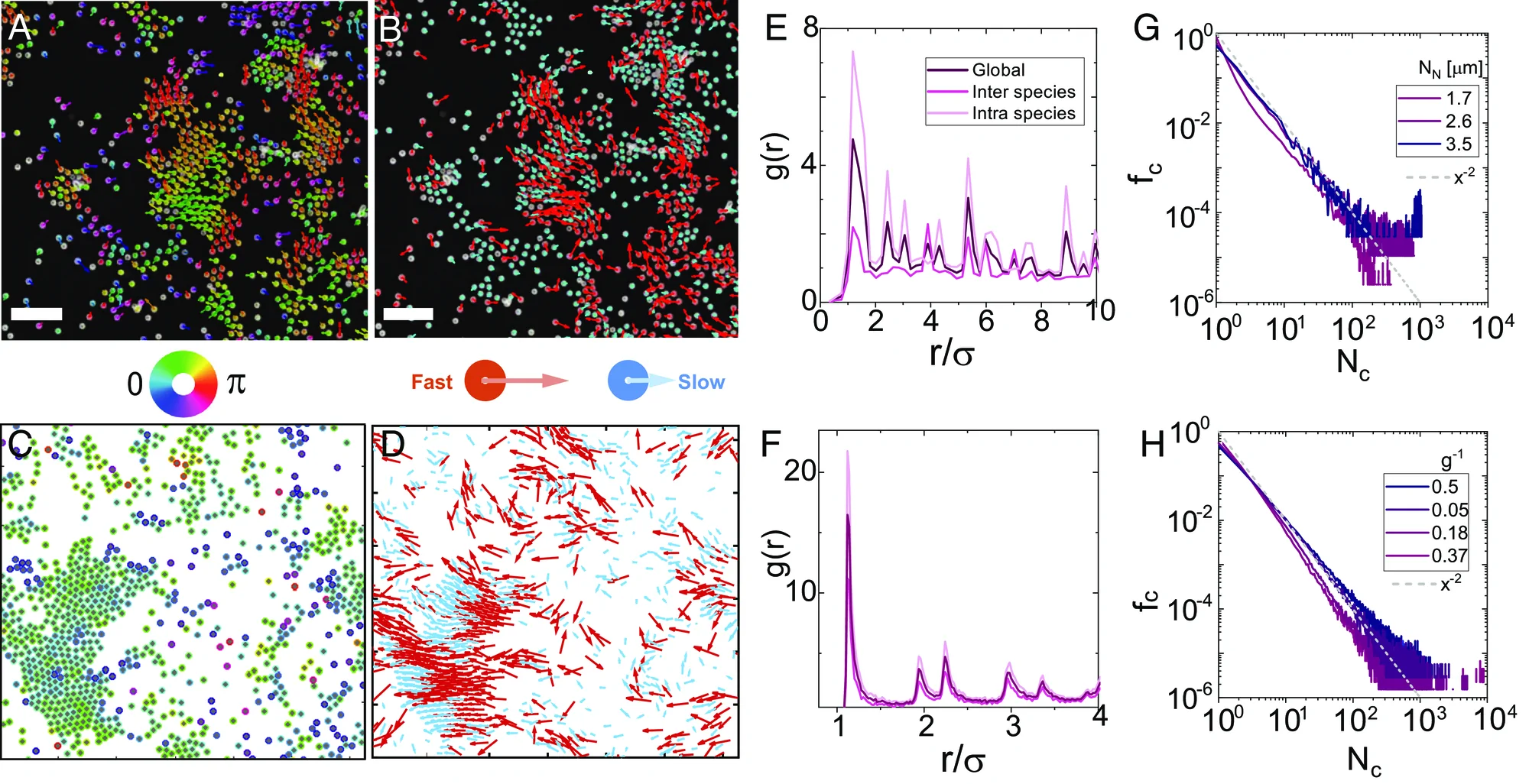
Segregation and cluster structure analysis. Screenshots of (*A* and *B*), experiments at 8 $V_{\mathrm{pp}}$, 1 kHz; and (*C* and *D*) analogous simulations with $g^{-1}=0.18$, $Pe_{S}^{\mathrm{sim}}=3.3$, $Pe_{F}^{\mathrm{sim}}=13$. In (*A* and *C*) the color depicts the particle orientation θ as shown in the color wheel. In *A* the arrows depict the velocity vector. *B* and *D* Same snapshot as for *A* and *C* coloring the particles by populations, with slow (cyan) and fast (red) particles at area fraction $\Phi_{A}\approx 0.2$, with $x_{F,exp}\approx 0.45$ and $x_{F,sim}\approx 0.5$. (Scale bars, 20 μm.) (*E* and *F*) Pair correlation functions g(r) for intraspecies (light pink), interspecies (magenta), and total particle pairs (dark pink) for experiments (*Top*) and simulations (*Bottom*). (*G* and *H*) Cluster size distributions $f_{c}\left(N_{c}\right)$ from experiments (*Top*) (cut-offs $N_{N}=1.7,2.6,3.5\;\mu$m) and simulations (*Bottom*) varying g, using the first peak of g(r) as a cut-off distance to define a cluster t. Dashed line: $f_{c}∼N_{c}^{-2}$.

As both particles are experimentally indistinguishable under optical microscopy, we performed a dynamical classification based on their individual distribution of displacements (*SI Appendix*). Similarly, we compute the analogue scenario with the ABP model in the presence of the fast ($Pe_{F}^{\mathrm{sim}}=$13) and slow ($Pe_{S}^{\mathrm{sim}}=$3) species (within the range that qualitatively agrees with our experiments), and for $g^{-1}\approx$ 0.2. In Fig. 2*A*–*D*, we show representative snapshots of experimental and numerical active binary mixtures comprising fast $SiO_{2}$–Pd and slow $SiO_{2}$-Au Janus colloids (Movies S3 and S4 for experiments and simulations, respectively). In contrast with previous works using catalytic or light-driven Janus particles (24, 64), slow particles do not get trapped in clusters of fast ones. In fact, a spatial segregation between the two species is observed as previously reported for nonreciprocal Quincke rollers of different sizes and thus swimming speeds (40). Such organization is quantified by the analysis of the pair correlation function g(r), as shown in Fig. 2 *E* and *F*. We compute the g(r) considering only correlations between particles of the same species (being fast/fast or slow/slow), between particles of opposite species (fast–slow) and all particles without distinction between the species. Here we observe that particles of the same species are more likely to cluster together, evidenced by higher and sharper peaks in the g(r). Because the polar cluster phase is highly transient (with clusters continuously forming, merging, and fragmenting across a broad range of sizes), a detailed characterization of individual cluster lifetimes and kinetics is not statistically robust. Thus, we measure the normalized frequency of cluster size $f_{c}$ at various cluster sizes (Fig. 2 *G* and *H*). We observe a rather broad distribution of cluster sizes, with a decay of $\propto N_{c}^{-2}$, meaning that the system is populated by clusters of any size, with no typical length scale.

The corresponding ABP dry model reproduces this cluster, whose statistics support the view that the observed clustering is controlled primarily by effective alignment and steric interactions, regardless of the system-specific electrohydrodynamic details. This agreement motivates the use of such a minimal model to analyze particle–particle interactions and their impact on transport and clustering in the following sections.

Within the ABP description, reducing *DR* at fixed alignment strength increases *g*, and would therefore be expected to stabilize polar order and produce longer-lived clusters. Experimentally, however, *DR* cannot be varied independently in the present system, since changes in viscosity, temperature, particle size, or wall coupling would also modify the electrohydrodynamic propulsion and alignment interactions. Accessing this regime would therefore require reestablishing the frequency- and voltage-dependent collective phase diagram.

## Cooperative Behavior in Effectively Nonreciprocal Species

**Fig. 3.**
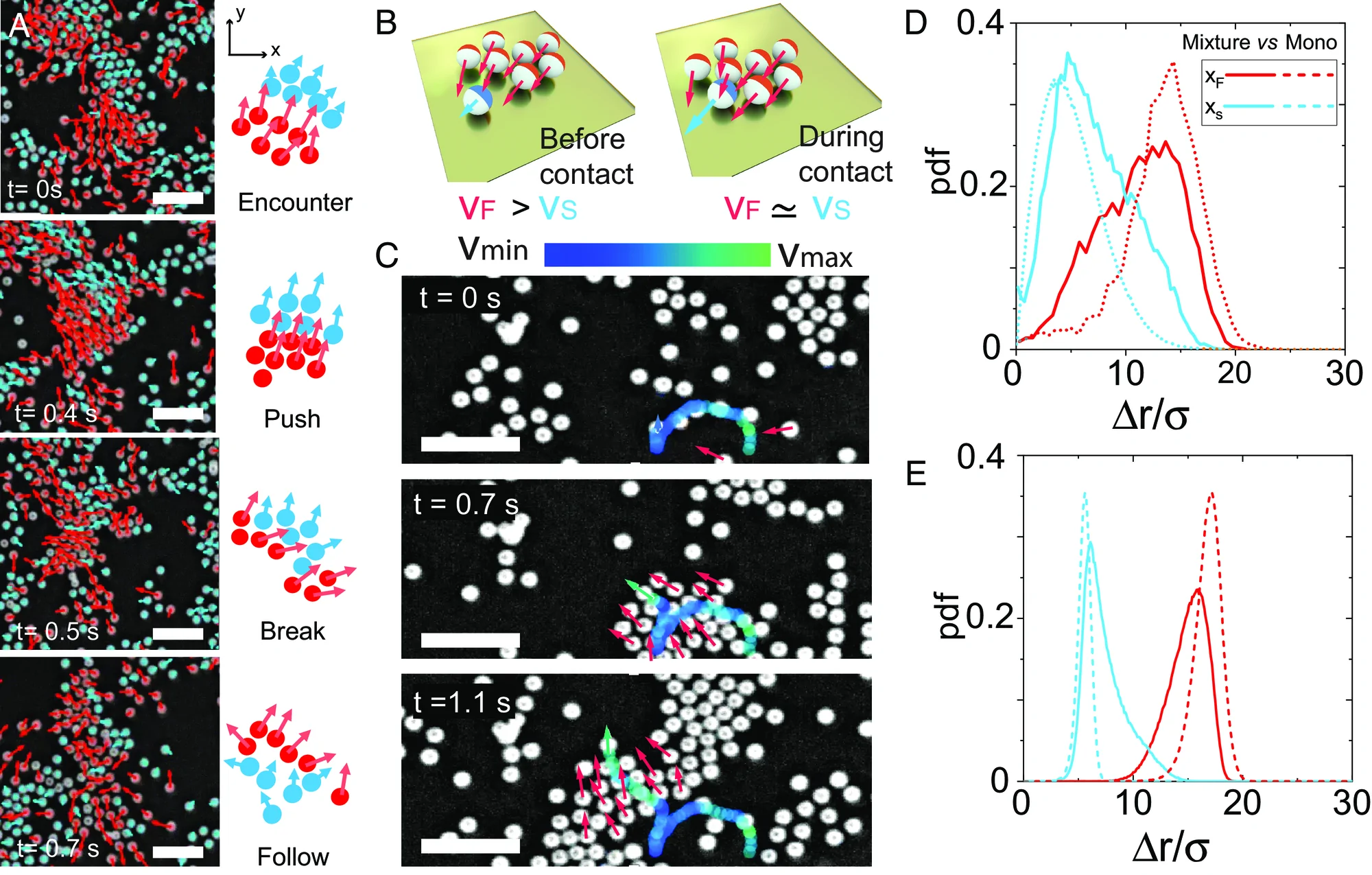
Interacting fast and slow clusters with cooperative behavior. (*A*) Screenshot of fluorescent movies of a cluster of slow (cyan) and fast (red) particles interacting with their corresponding schematic representation (*Right*). The cluster interaction follows four main stages: i) encounter of a fast cluster and a slow cluster, ii) pushing of the slow cluster, iii) breakage of the cluster, and iv) slow particles trying to follow the fast particles. (Scale bars, 20 μm.) (*B*) Schematic of a cluster of fast particles before (*Left*) and during (*Right*) collision with a slow particle (or cluster of slow particles), showing the slow particle being pushed by a fast-particle cluster, resulting in enhanced velocity of the slow particle. The arrow depicts the velocity vector for fast (red) and slow (cyan). (*C*) Corresponding screenshots from fluorescence movies of a slow particle being pushed by a cluster of fast particles. The trajectory color represents the instantaneous velocity, from slow (dark blue) to fast (light blue). (Scale bars, 20 μm.) Displacement distributions at t=0.3 s from (*D*) experiments (*E*) Simulations for slow $Pe_{S}^{\mathrm{sim}}=3$ ($x_{S}$, cyan) and fast $Pe_{F}^{\mathrm{sim}}=13$ ($x_{F}$, red) particles in mixtures (solid) and monodisperse controls (dashed).

We further study the interaction between both cluster populations and their dynamical behavior. Overall, we consistently find interesting collective phenomena: dense swarms mainly composed of fast particles push slow particles from the back. Experimentally, most cluster encounters follow this reproducible sequence (Fig. 3*A*). The collision of two clusters leads to the fast polar cluster pushing of the slow cluster at the impact front. This “push” stage produces a net displacement and alignment of the slow aggregate in the direction of the fast clusters’ polarity. This is directly visible in individual slow-particle trajectories during encounters, where their velocity increases while they are dragged by the fast cluster (Fig. 3 *B* and *C* and *SI Appendix*, Fig. S5 and Movie S5). The pushing stage is typically followed by a rapid loss of cohesion in the slow cluster, leading to fragmentation into smaller subclusters and isolated particles. After the breakup, a fraction of the slow particles remains transiently entrained at the rear of the fast cluster and attempts to follow its motion, while the rest lags behind and breaks completely to form other slow clusters. This regime differs from previous experimental active mixtures involving either active populations with different sizes under confinement (40) or active–passive mixtures (30). Here, both populations are active, have the same size and comparable rotational diffusion, and interact through transient polar-cluster encounters that modify the dynamics of both species.

Although at the microscopic level, the interactions between two different active particles might fulfill the action–reaction principle, nonreciprocity generically emerges at the mesoscale level from the mere combination of steric forces and distinct self-propulsion velocities (or persistence times), as explained in detail in *Materials and Methods* (31). In the overdamped regime relevant here, two species that self-propel at different speeds generically couple through an effective friction/mobility that is asymmetrical. In other words, nonreciprocity is therefore an underlying, generic feature of dissipative active mixtures with heterogeneous propulsion. In our experiments, the effective nonreciprocity between both species manifests primarily at the level of cluster–cluster interactions, i.e., in the sequence and directionality of encounter outcomes. Such traveling clusters with one species chasing the other is a fingerprint of non-reciprocity, reminiscent of predator–prey dynamics (65, 66). Note that this morphology has a purely dynamical origin, since in this regime, the onset of polar order is at the same parameter value for both slow and fast particles.

The “pushing” or local cooperation effect is quantitatively captured by the overall displacement distributions for each species in the mixture, depicted in Fig. 3 *D* and *E* for experiments and numerical simulations, extracted for mixtures at area fraction $\Phi_{A}\approx 0.2$ with $x_{F}\approx 0.5$. Compared to the monodisperse suspensions, the bidisperse distributions develop pronounced asymmetric tails, indicating intermittent events beyond single-species fluctuations. The slow species displays an excess probability at large displacements: a residual high-displacement tail consistent with transient advection by fast polar clusters, such that slow particles occasionally move faster than in the corresponding monodisperse case. Conversely, the fast species develops a low-displacement tail, consistent with episodes of slowing down when fast clusters engage with slow aggregates (*SI Appendix*, Fig. S6). Overall, these tails provide a statistical signature of short-lived interspecies coupling mediated by clustered encounters, rather than a homogeneous change of mobility at the ensemble level. Importantly, these distributions do not provide a direct metric of nonreciprocity: the displacement tails of the two species can be of comparable magnitude, and the PDFs do not appear strongly asymmetric. Instead, relative to the monodisperse suspensions, the PDFs develop broader tails consistent with intermittent, contact-driven events beyond single-species fluctuations, in line with the encounter sequence described above. As a reference, we also quantify the swimming speed of the slow and fast particles using the distribution of displacements as a function of low and dense $\Phi_{A}$ (*SI Appendix*, Fig. S7). As expected, we see a decrease in the mobility in the dense phase with respect to the diluted phase, mainly induced by crowding effects (63). To quantify this response after cluster encounters at the level of individual trajectories, we compare the characteristic velocity of the same particle in the free and clustered states, for slow and fast particles (*SI Appendix*, Fig. S8). The two populations respond in opposite directions and with unequal relative magnitudes to clustered environments. This particle-wise response asymmetry provides an experimental coarse-grained signature of the asymmetric dynamics emerging from motility contrast.

In our simulations, the effect of individual polar clusters interaction and cooperation is clearly present. Although the ABP model does not include any explicit nonreciprocal pair interaction, differences in self-propulsion speed generate nonreciprocal dynamical responses. In the model, g controls the formation and stability of the polar-cluster phase. Once this phase is established, the motility contrast between the two populations drives segregation, transient modification of the dynamics of both populations, and the asymmetric fast–slow response underlying the catch-and-run dynamics. This suggests that the observed asymmetry can be an intrinsic property of poly-disperse active systems with mixed-motility: differences in self-propulsion set unequal momentum fluxes and collision rates, while alignment stabilizes polar aggregates that act as “carriers” able to advect the slower ones. In this sense, nonreciprocity need not be imposed microscopically, as it arises from coarse-graining the dynamics of mixtures. Because clusters are short-lived and continuously form and break, the “fast-push” cooperation is primarily a local, intermittent effect. It does not necessarily translate into a sustained enhancement of mobility at the ensemble level, since spatial and temporal averaging washes out these transient events.

**Fig. 4.**
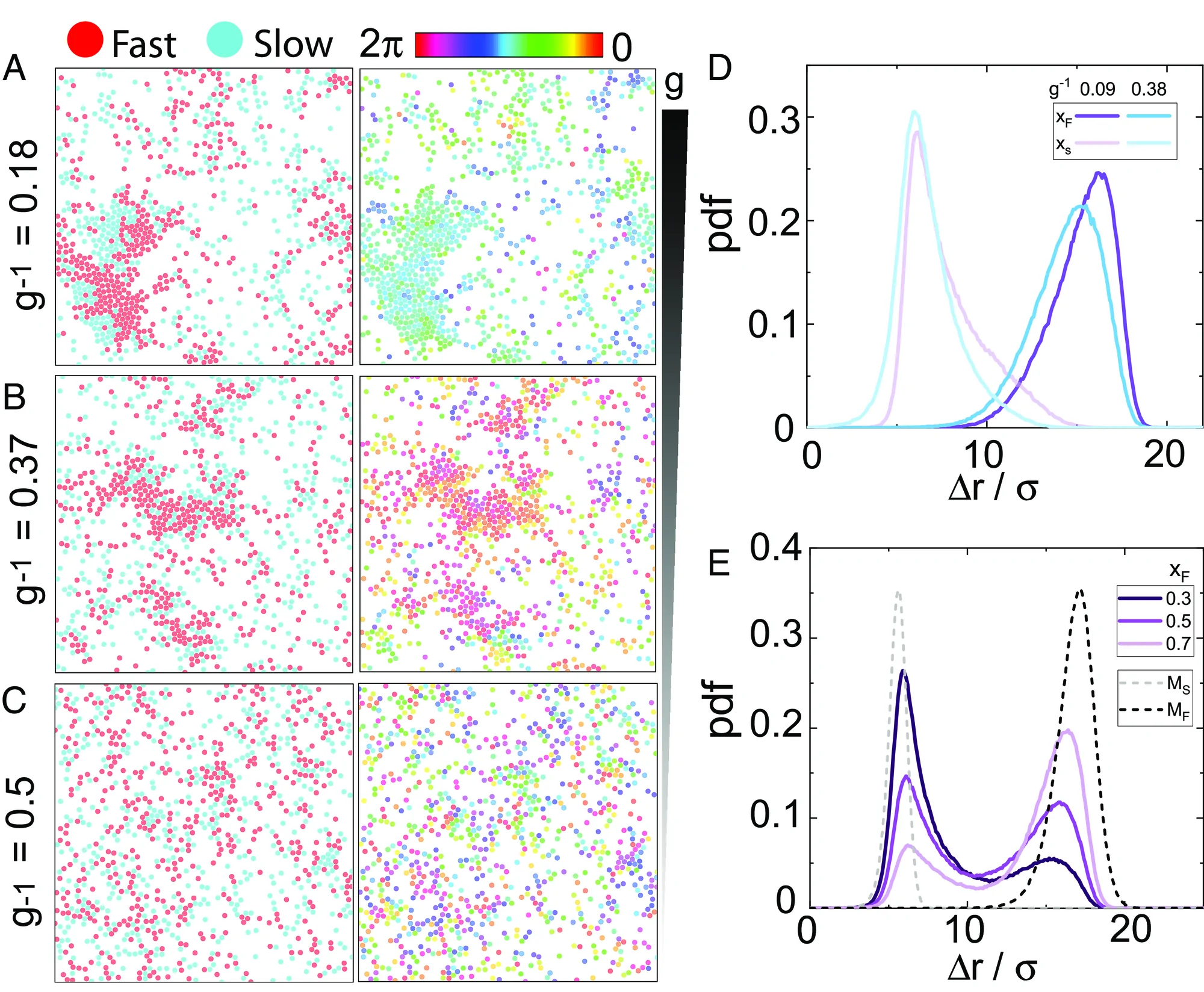
Simulation of binary mixtures of active particles as a function of alignment strength. Snapshots from simulations with two species with different alignment strength (*A*) $g^{-1}=0.18$, (*B*) $g^{-1}=0.37$, and (*C*) $g^{-1}=0.5$. The fast particles ($Pe_{F}^{\mathrm{sim}}=13$) are in red and the slow ones ($Pe_{S}^{\mathrm{sim}}=3$) in cyan (left column). The graphs in the right column show the corresponding particle orientations. In all the simulations $x_{F}=0.5$ and $\Phi_{A}\approx 0.2$. (*D*) PDF of displacements from simulations at fixed particle ratio $x_{F}=0.5$, and varying the alignment force $g^{-1}$ for $g^{-1}=0.09$ (magenta) and $g^{-1}=0.38$ (blue). The transparent and solid lines indicate the slow species and fast species, respectively. (*E*) PDF of displacements from simulations for varying fraction of fast particles at fixed alignment $g^{-1}=0.18$ and varying species ratio $x_{F}=0.3$ (dark magenta), $x_{F}=0.5$ (magenta), and $x_{F}=0.7$ (light magenta). The dashed lines represent the monodisperse case for slow (transparent) and fast (solid) particles. In these simulations PDF $Pe_{F}^{\mathrm{sim}}=13$, $Pe_{S}^{\mathrm{sim}}=3$, and $\Phi_{A}\approx 0.2$ total particle density.

As it is experimentally not possible to control the initial location of slow and fast particles, nor the in-out flux of both species within the region of interest while acquiring the videos, systematically studying how the ratio of species influences the cooperative behavior is beyond our experimental capabilities. However, we can fully explore the effect of species ratio using numerical simulations for different values of $g^{-1}$, for the Peclet numbers used in this study in simulations (Fig. 4*A*–*C*). The latter is crucial for the segregation of both phases and the positioning of the populations with respect to each other. In Fig. 4*D*, we show the effect of alignment strength in binary mixtures of 1:1 ratio of species. Here, we always observe the emergence of a polar cluster phase (with high local ordering) above a certain threshold of alignment, where the fast particles always push groups of slow particles, which are eventually left behind or disassembled. This is in stark contrast with previous experimental and numerical works done in the regime of motility-induced phase separation, where slow or passive particles get trapped within clusters of the fast ones. The effect of alignment strength seems to affect mostly the slowest particles with residual tails shifting toward higher displacements. In addition, the ratio between each species determines the overall behavior of the mixture, based on the interspecies interaction, and thus their relative displacements (Fig. 4*E*). Not surprisingly, we see an overall effect on the mixture led by the dominant population: when the majority of particles are fast, the slow particles tend to become faster, and vice versa. Not only is there a slight shift in the characteristic peak of the distribution of displacements affecting their relative speeds, but the distribution becomes biased and exhibits a valley that shows intermediate behavior, as previously shown when compared to the experimental conditions. This is not present in the monodisperse case. Thus, changing $g^{-1}$, the mean peak position of the displacements can be shifted (the motility of each species is affected), while the effect of the fraction of the populations controls the weight of each population, where one species dominates over the other one (Movie S6).

## Conclusion and Outlook

Here, we demonstrate how to engineer binary mixtures of active colloids by exploiting the interplay between particle material properties and external AC electric fields. We fabricate two types of Janus particles with distinct motilities yet similar rotational diffusion. Motility contrast leads to distinct clustering behavior, differential participation in dense phases, and spatial domain formation not captured by simple single-component active matter models. Effective nonreciprocal couplings thus emerge at the coarse-grained level, meaning that one species responds in a nonsymmetric way to the presence of the other one, even though microscopic interactions are symmetric.

Our results reveal that the two species of particles can self-organize into polar clusters of fast- and slow-moving species at given particle densities. The interactions between fast and slow particles lead to segregation and cooperative dynamics, with fast polar clusters locally advecting and transiently accelerating slow particles. Our experimental observations are supported by numerical simulations using a simple model of active Brownian particles with varying alignment strength, which effectively captures the essential features of the observed behavior without explicit electrohydrodynamic effects or nonreciprocity. The model provides a framework to map the role of alignment strength and particle density, leading to a deeper understanding of the conditions under which collective behavior emerges. As this is purely material-dependent (67), the swimming behavior and potential interactions can be tuned using different dielectric materials and metals to fabricate and design rich collective phenomena in Janus particle mixtures.

The ability to fine-tune these interactions opens up new possibilities for designing reconfigurable active materials. Such control over particle behavior and interactions could be interesting for active materials with mechanical properties that depend on their internal structure, as previously proposed in active *fractalytes* (68). Moreover, the intrinsic differences in motility and flow fields around the particles could be exploited as two-species model systems with nonreciprocal interactions (43). More generally, tuning rotational diffusion offers a route to engineer the order and lifetime of polar clusters, although this requires accounting for the concomitant changes in electrohydrodynamic propulsion and interactions. An interesting next step is to test such mixtures in structured landscapes (e.g., Gaussian wells), where fast-driven pushing could bias the escape of slow particles from traps or promote directed transport.

## Materials and Methods

### Fabrication of Janus Particles.

To fabricate our active Janus particles, silica colloids with 2.1 μm diameter (5% w/v, microParticles GmbH, Germany) are diluted to 1:6 in MilliQ water and spread on a glass slide, previously made hydrophilic by a 2-min air plasma treatment. Upon drying the suspension, a close-packed particle monolayer is formed. The monolayer is then sputter-coated (Safematic CCU-010, Switzerland) with 2 nm Cr for both particles, a second layer with 4 nm of Au for one type of particle (slow), and 6.5 nm of Pd for the other particle (fast). The Cr layer is used to promote the adhesion of the other metals to silica. The particles are retrieved from the glass slide by sonicating the slide containing the coated monolayer in MilliQ water. For the bidisperse experiments, we prepare a stock solution using the same volume of the Pt-$SiO_{2}$ and Au-$SiO_{2}$ particle in MilliQ water (conductivity $\sigma_{m}=5\;\times \;10^{-5}$ Sm^−1^).

### Cell Preparation.

The transparent electrodes are fabricated from 18 mm × 18 mm No. 0 cover glasses (85 to 115 μm-thick, Menzel Gläser, Germany) covered with 3 nm of Cr and 10 nm of Au, deposited by metal evaporation (Evatec BAK501 LL, Switzerland), followed by 10 nm of SiO_2_, deposited by plasma-enhanced chemical vapor deposition (STS Multiplex CVD, UK) to minimize the adhesion of particles to the substrate during the experiments. A water droplet containing the particles is deposited on the bottom electrode within the 9 mm-circular opening of a 0.12 mm-thick sealing spacer (Grace Bio-Labs SecureSeal, USA). The top and bottom electrodes are connected to a signal generator (National Instruments Agilent 3352X, USA) that applies the AC electric field, varying frequencies from 0.8 to 5 kHz and varying the voltage between 1 and 10 $V_{\mathrm{PP}}$.

### AC Electric Field Experiments.

The Janus particles are imaged with a Zeiss Axio microscope in transmission, and image sequences are taken with a sCMOS camera (Andor Zyla 4.2) at varying frame rates between 10 and 100 fps, depending on the swimming speed and concentration of the Janus particles, with a variable field of view among experiments. The positions of the center of the JPs and the metal caps are located using Matlab routines. Then, a vector connecting both centers is used to determine the orientation of the particle at each frame. In addition, the center of mass of the particle is used to determine the velocity vector. Image series of the metallic Janus particles actuated by the AC electric fields are acquired with a 20× and a 40× objective (Zeiss).

### Particle-Based Model.

We consider a system of self-propelled particles in a two-dimensional square box of side L with periodic boundary conditions (PBC). The equations governing the dynamics of the system are the coupled overdamped Langevin equations,

[2] $$\begin{array}{c} {\dot{r}}_{i}=v_{0}^{\alpha }n_{i}+\mu F_{i}+\sqrt{2D_{0}}\eta_{i}, \end{array}$$

[3] $$\begin{array}{c} {\dot{\varphi }}_{i}=J\sum_{j\neq i}v\left(r_{\mathrm{ij}}\right)\text{sin}\left(\varphi_{j}-\varphi_{i}\right)+\sqrt{2D_{r}}\nu_{i}. \end{array}$$

Each particle i∈{1,...,N} self-propels at constant speed, $v_{0}^{\alpha }\in \left\{v_{0}^{F},v_{0}^{S}\right\}$, where $v_{0}^{F}$ is the nominal speed of the fast species, and $v_{0}^{S}$ of the slow one. $\eta_{i}$ and $\nu_{i}$ are two independent white Gaussian noises with zero mean and unit variance: $\left\langle \eta_{i}^{\alpha }\left(t\right)\eta_{j}^{\beta }\left(t^{\prime }\right)\right\rangle =\delta_{\mathrm{ij}}\delta^{\alpha \beta }\delta \left(t-t^{\prime }\right)$ and $\left\langle \nu_{i}\left(t\right)\nu_{j}\left(t^{\prime }\right)\right\rangle =\delta_{\mathrm{ij}}\delta \left(t-t^{\prime }\right)$ (α, β denoting Cartesian coordinates). Rotational noise sets a characteristic time scale, given by the inverse of the rotational diffusion coefficient, $\tau =D_{R}^{-1}$, the persistence time, and a characteristic length scale given by $l_{p}=v_{0}\tau$, the persistence length. The noise $\eta_{i}$ mimics a thermal bath surrounding the particles at temperature T. The diffusivity $D_{0}$ and mobility μ fulfill the Einstein relation $D_{0}=\mu k_{B}T$.

Steric interparticle forces, $F_{i}=-\sum_{j\neq i}\nabla_{i}u\left(r_{\mathrm{ij}}\right)$, derive from a WCA potential,

[4] $$\begin{array}{c} u\left(r_{\mathrm{ij}}\right)=\left\{\begin{array}{cc} 4u_{0}\left[{\left(\frac{d}{r_{\mathrm{ij}}}\right)}^{12}-{\left(\frac{d}{r_{\mathrm{ij}}}\right)}^{6}\right]+u_{0} & r_{\mathrm{ij}}\leq R\\ 0 & r_{\mathrm{ij}}>R, \end{array}\right. \end{array}$$

where $r_{\mathrm{ij}}=\left|r_{i}-r_{j}\right|$. The cutoff distance is $R=2^{1/6}d$ and $u_{0}$ corresponds to the characteristic energy scale of the potential.

Aligning torques are introduced in terms of the sum of the phase difference, as in models of ferromagnets, which runs over the particle’s vicinity, defined by a spatially decaying function

[5] $$\begin{array}{c} v\left(r_{\mathrm{ij}}\right)=\left\{\begin{array}{cc} \frac{2}{\pi R_{\varphi }^{2}}{\left(r_{\mathrm{ij}}-R_{\varphi }\right)}^{2} & r_{\mathrm{ij}}<R_{\varphi }\\ 0 & r_{\mathrm{ij}}>R_{\varphi }, \end{array}\right. \end{array}$$

where the cutoff distance $R_{\varphi }$ sets the interaction range beyond which particles do not align. The strength of the aligning torques is controlled by the coupling parameter J≥0, which mimics an alignment mechanism of nonsteric origin, in the sense that it is decoupled from the particles’ shape.

### Brownian Dynamics Simulations.

We choose the particles’ diameter σ=1 to be the unit of length, $\tau_{0}=\frac{d^{2}}{D_{0}}=1$ the unit of time and $k_{B}T=1$ the unit of energy. The strength of the pairwise repulsive interaction is $u_{0}=100k_{B}T$. The thermal and rotational diffusion coefficients fulfill $D_{0}=\frac{d^{2}D_{R}}{3}$, which leads to a fixed rotational diffusion coefficient $D_{R}=3/\tau_{0}$. The alignment cut-off distance is set to $R_{\varphi }=2d$, and therefore the particles need to be in contact to align their directions of self-propulsion. We identify the dimensionless parameter $g=\frac{2J}{D_{R}\pi R_{\varphi }^{2}}$, which accounts for the strength of the alignment interaction compared to the rotational diffusion and the Péclet number, $\text{Pe}=\frac{v_{0}}{dD_{R}}=\frac{l_{p}}{d}$, which quantifies the persistence of the particle’s motion. The global packing fraction is $\phi =\frac{N\pi d^{2}}{4L^{2}}$ (denoted $\Phi_{A}$ in experiments).

In binary mixtures, the ratio between the fast and the slow self-propulsion speed is defined as $v_{0}^{F}=f_{v}v_{0}^{S}$. In addition to the speed of self-propulsion, the rest of the parameters are identical for both species. The fraction of fast particles is $x_{F}=N_{F}/N$, and the fraction of slow ones is, correspondingly, $x_{S}=1-x_{F}$. We thus have a parameter space comprising $\left(\phi ,g,\;{\text{Pe}}^{S},f_{v},x_{F}\right)$, where ${\text{Pe}}^{S}$ stands for the Péclet number of the slow species.

The Langevin equations defining the model are integrated using an Euler–Maruyama scheme, employing a timestep $\Delta t=10^{-4}$. Results are computed by averaging over 1,000 independent configurations in the stationary state.

### Effective Nonreciprocal Response between Active Particles with Different Self-Propulsion Speeds.

Let us consider two different self-propelled particles interacting via a conservative pairwise force and velocity alignment as described by Eq. **2**. The orientation difference $\delta =\varphi_{2}-\varphi_{1}$ between them follows

[6] $$\begin{array}{c} \dot{\delta }=-2Jv\left(r\right)\sin\delta +\sqrt{4D_{r}}\nu , \end{array}$$

where ν is a Gaussian white noise with zero mean and unit variance. The steady-state in the absence of noise corresponds to the fixed point δ=0. The translational dynamics can thus be written, up to first order in δ as

[7] $$\begin{array}{c} {\dot{r}}_{1}=v_{0}^{1}n+\mu F_{1}-\frac{1}{2}v_{0}^{1}\delta u+\sqrt{2D_{0}}\eta_{1}, \end{array}$$

[8] $$\begin{array}{c} {\dot{r}}_{2}=v_{0}^{2}n+\mu F_{2}+\frac{1}{2}v_{0}^{2}\delta u+\sqrt{2D_{0}}\eta_{2}, \end{array}$$

where

[9] $$\begin{array}{c} n_{1}=n-\frac{1}{2}\delta u\;,\;n_{2}=n+\frac{1}{2}\delta u \end{array}$$

with n=(cosϕ,sinϕ), $\phi =(\varphi_{1}+\varphi_{2})/2$, and u=(−sinϕ,cosϕ) (π/2-rotation of n). Then, one can easily see that an orientation mismatch generates unequal responses on the two particles as soon as $v_{0}^{1}\neq v_{0}^{2}$, since

[10] $$\begin{array}{c} \frac{\partial {\dot{r}}_{1}}{\partial \delta }=-\frac{v_{0}^{1}}{2}u\;,\;\frac{\partial {\dot{r}}_{2}}{\partial \delta }=\frac{v_{0}^{2}}{2}u. \end{array}$$

In this sense, the model can be considered nonreciprocal.

## Supplementary Material

Appendix 01 (PDF)

Movie S1. Experimental movie of a semi-dense phase (Φ_A_ ≈ 0.15) of fast particles transitioning from elastic collisions (2 kHz, g^−1^ > 1) to polar alignment (1 kHz, g^−1^ < 1) at a fixed voltage 8 *V_pp_*.

Movie S2. Numerical movie depicting the effect of Pe at a fixed g^−1^ =0.2. The colours indicate the individual particle velocity orientation.

Movie S3. Experimental binary mixtures movies at Φ_A_ ≈ 0.2 at 1 kHz and 8V_pp_. The movie on the left depicts the population coexistence with the slow (blue) and fast (red) (x_F_ ≈ 0.47). The arrows indicate the magnitude of the velocity. The movie on the right is the corresponding representation of the velocity vector magnitude and orientations in different colours.

Movie S4. Numerical binary mixtures movies at Φ_A_ ≈ 0.2 at g^−1^ =0.2, as analogous to Movie S2. The movie on the left depicts the population coexistence with the slow (blue) and fast (red) with Pe 3.3 and 13.3, respectively (x_F_ ≈ 0.5). The movie on the right is the corresponding representation, where the colours represent the particle velocity orientation.

Movie S5. Movie of an event of a single slow particle being pushed by a fast cluster. The coloured trajectory depicts the instantaneous velocity of the slow particle (blue) at 1 kHz and 8V_pp_.

Movie S6. Numerical experiments for binary mixtures at Φ_A_ = 0.2 for fixed alignment g^−1^ =0.2 at varying Pe.

## Data Availability

Raw videos and figure data have been deposited in FigShare (69).
