# Supplementary material for: Cooperation in binary mixtures of active colloids
Source: Proc Natl Acad Sci U S A. 2026 Oct 6;123(41):e2609894123. doi: 10.1073/pnas.2609894123 (PMC13663720; doi:10.1073/pnas.2609894123)
Supplement: Supplementary file 1 [file pnas.2609894123.sapp.pdf]

Supporting Information for

Cooperation in binary mixtures of active colloids

Laura Alvarez, Elena Sesé-Sansa, Demian Levis, Ignacio Pagonabarraga and Lucio Isa

E-mail: laura.alvarez-frances@u-bordeaux.fr, levis@ub.edu

This PDF file includes:

Supporting text
Figs. S1 to S8
Legends for Movies S1 to S6

Other supporting materials for this manuscript include the following:

Movies S1 to S6

Supporting Information Text

Fabrication of Janus particles with different Pe

Given that the Janus sphere main component is the same SiO_2 sphere, we have taken into account the density of each metal sputter onto the SiO_2 to match the masses and avoid heavy-bottom effects that might affect the dynamics of the particles. For this, we calculate the mass associated with a thin cap around the SiO_2 sphere, taking into account both the densities for Pd ($\rho_{\mathrm{Pd}} = 12\mathrm{gcm}^{-3}$) and Au ($\rho_{\mathrm{Au}} = 19.3\mathrm{gcm}^{-3}$). In particular, we first calculate the volume of the cap as

$$V_{cap} = \frac{2}{3}\pi R^3 - \frac{2}{3}\pi r^3 \qquad [1]$$

where R is the total radius of the sphere including the cap, and r is the internal radios of the sphere accounting the thickness of the cap (Fig.S1). We then calculate the mass of caps of different thicknesses using the different densities of Au and Pd, aiming for the same mass using $\mathrm{m_{cap}} = \mathrm{V_{cap}} * \rho$. In Fig.S1 we show two SEM images of each type of particle with a fake colour code for distinction.

1. Dynamical characterization of bidisperse dense mixtures

To classify particles as fast or slow in the binary mixtures when optical distinction is not possible, we analyze full single-particle trajectories and compute cumulative in-plane displacements separately in the free and clustered states. We retain only trajectories containing at least 300 detected positions and sampling both free and clustered states during the acquisition time. For each particle i, we define

$$r^{\alpha}_{xy,i} = \sum_{k\in\alpha} \|\mathbf{r}_i(t_{k+1}) - \mathbf{r}_i(t_k)\|_{xy}\,, \qquad \alpha \in \{\mathrm{free}, \mathrm{clust}\}, \qquad [2]$$

where $r^{\alpha}_{xy,i}$ is the cumulative path length in the xy-plane over trajectory segments assigned to state α. We then construct the corresponding population distributions $P(r^{free}_{xy})$ and $P(r^{clust}_{xy})$.

To minimize state-sampling bias, we retain only trajectories in which particles visit both free and clustered states during the acquisition window, so that mobility estimates are not dominated by a single environment. The local state is determined from the number of neighbouring particles ($N \geq 4$) within a radial cut-off distance R. A particle is assigned to the clustered state when more than four neighbours are detected within this radius, and to the free state otherwise. To suppress isolated fluctuations in the local neighbour count, the state must persist for at least 5 consecutive frames (Δt = 0.15 s, for a frame rate of 30 fps). In the free state, fast particles exhibit larger r_{xy} than slow particles. In the clustered state, fast particles show reduced r_{xy} due to confinement and interactions with neighbouring particles, whereas slow particles show increased r_{xy}, consistent with their forcing and entrainment by fast-particle clusters. The distinction between the two species is therefore obtained from the complete trajectory-level dynamical fingerprint and not from an instantaneous velocity or an individual collision event. To quantify whether the difference between the two states remains significant at the level of individual trajectories, we additionally perform a paired particle-wise analysis. For each particle i, we calculate its characteristic velocity in the free and clustered states, $v_{i,free}$ and $v_{i,cluster}$, and define

$$\Delta v_i = v_{i,cluster} - v_{i,free}. \qquad [3]$$

The distributions shown in Fig. S8e are constructed from one paired velocity difference per particle, so that each trajectory serves as its own reference. This analysis is not used to assign particle identity, but rather to verify that state-dependent changes (being free or in a cluster interacting with another cluster) are sufficiently large within individual trajectories, so the dynamical classification can be done. Slow particles exhibit a median velocity increase of $+7.8\ \mu m\, s^{-1}$, whereas fast particles exhibit a median decrease of $-10.3\ \mu m\, s^{-1}$.

We also calculate the corresponding relative change,

$$\Delta v_i^{rel} = 100\frac{v_{i,cluster} - v_{i,free}}{v_{i,free}}. \qquad [4]$$

The inset of Fig. S8e shows median relative changes of +24.4% for slow particles and −16.1% for fast particles. Thus, although the absolute velocity change is larger for the fast population, the proportional response is stronger for the slow population.

Because trajectory durations and state residence times are finite and not perfectly balanced, a small residual under- or over-estimation may remain in the class-resolved displacement values. We therefore treat this as a systematic uncertainty and verify that the qualitative population-level trends are robust to reasonable variations in the state-occupancy filters and classification threshold. On average, fast particles exhibit smaller displacements in the clustered state than in the free state, whereas slow particles exhibit larger displacements in the clustered state.

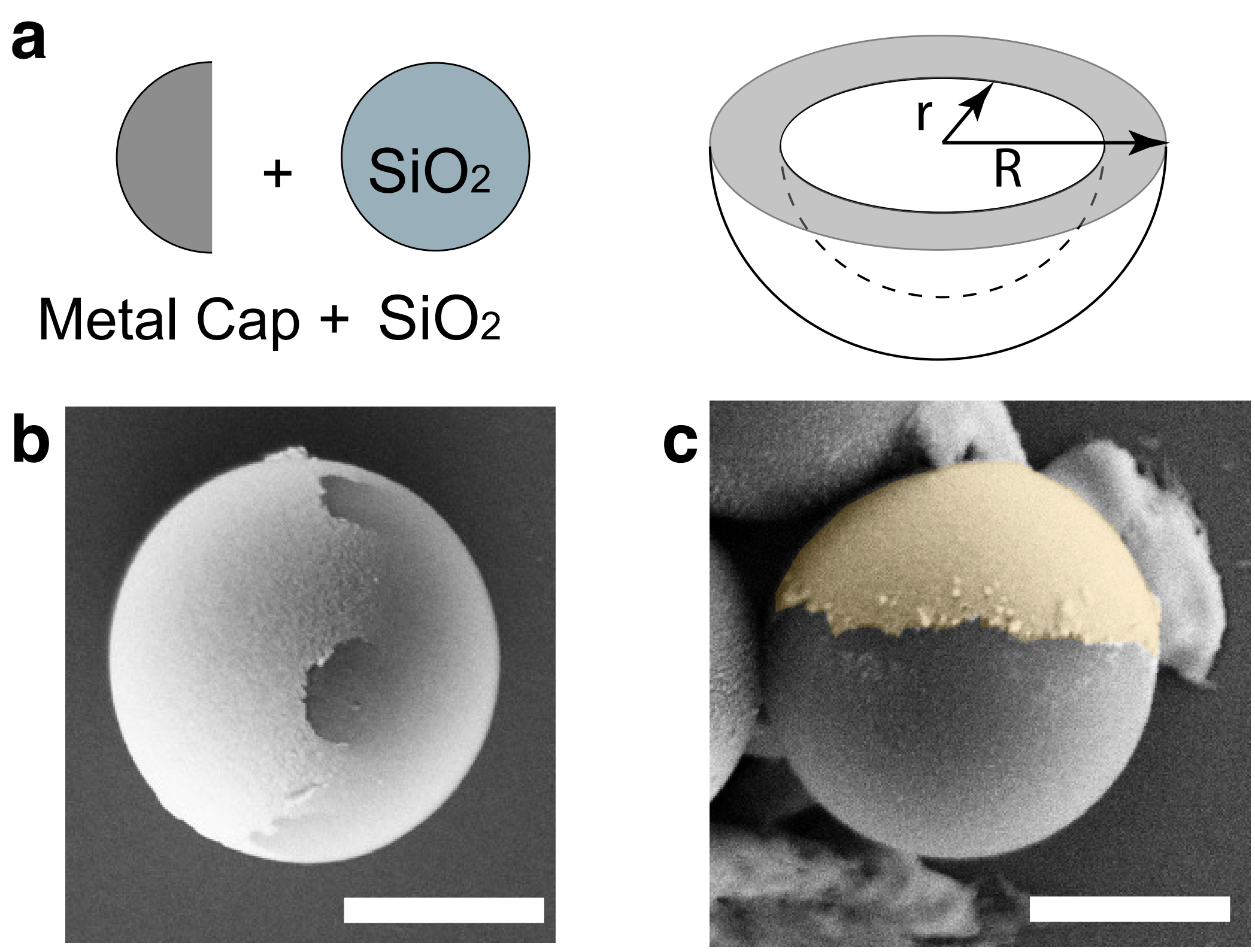

Fig. S1. **a**, Scheme of the Janus sphere composed by a metal cap and a $\mathrm{SiO_2}$ particle, with the corresponding scheme of the cap, being r the inner radius and R the total radius of the cap sputtered on the sphere. Scanning electron microscopy images of **b**, a $\mathrm{SiO_2-Pd}$ and **b**, $\mathrm{SiO_2-Au}$ Janus particles. The $\mathrm{SiO_2-Au}$ is artificially coloured. The scale bar depicts 1.1 $\mu\mathrm{m}$

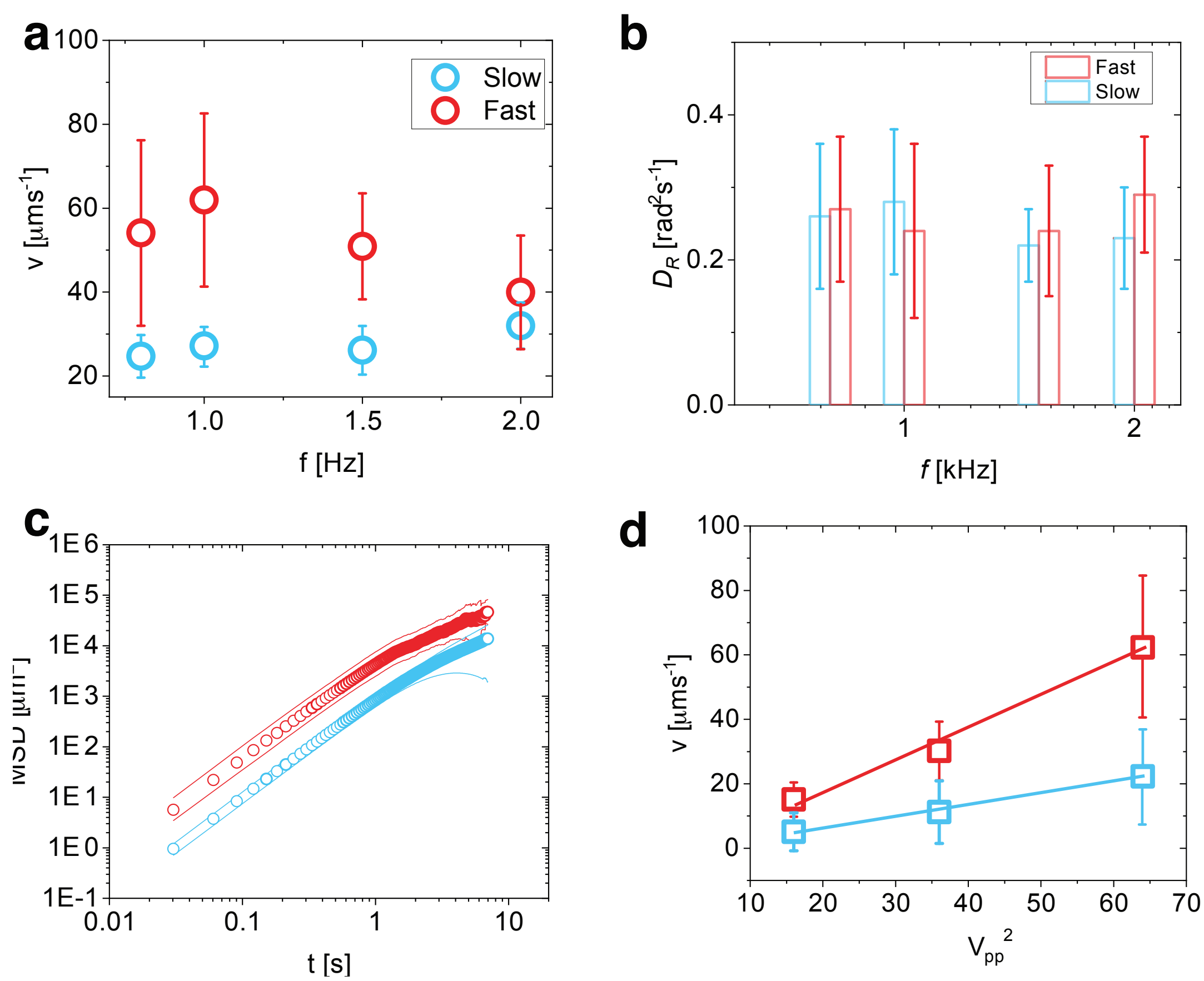

Fig. S2. **a**, Velocity as a function of frequency (kHz) at a fixed voltage (8 $\mathrm{V_{pp}}$) for the fast $\mathrm{SiO_2 - Pd}$ (red) and slow $\mathrm{SiO_2 - Au}$ (cyan) Janus particles, obtained from individual fitting of the short-time mean-squared displacements for more than 1000 trajectories for each species in diluted conditions $\Phi_A \approx 0.01$. The error bar depicts the standard deviation within a 95% confidence interval. **b** Rotational diffusivity $\mathrm{D_R}$ as a function of frequency for fast and slow particles, obtained from the long-time fitting of the mean-squared displacement in log-log, as $\mathrm{D_R^{-1}} = \tau_\mathrm{R}$. **c** Mean-squared displacement at 1 kHz and 8 $\mathrm{V_{pp}}$for fast (red) and slow (cyan) Janus particles. The error bars are the standard deviation calculated from independent MSDs. **c**, Linear dependence of the velocity for fast (red) and slow (cyan) Janus particles as a function of the applied electric field squared at 1 kHz.

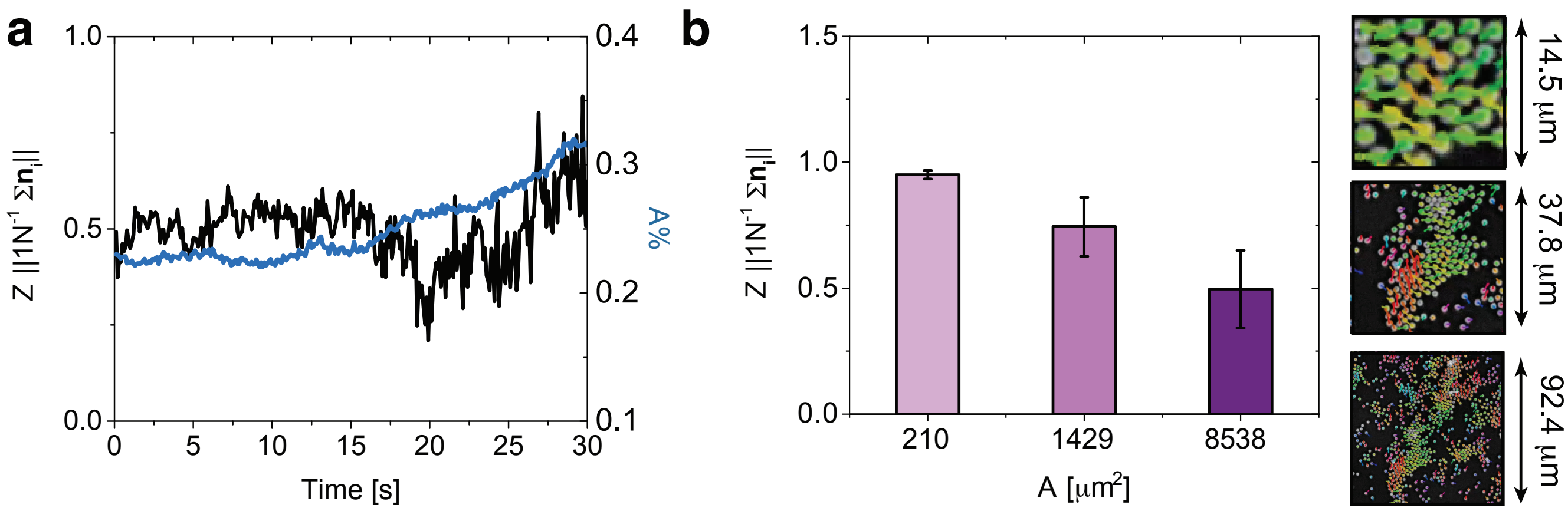

Fig. S3. **a**, Global order parameter Z and area fraction Φ_A as a function of time for Movie S4. **b** Examples of global order Z varying the region of interest (ROI) area, with the corresponding snapshot from experiments at 1 kHz, 8 $\mathrm{V_{pp}}$ and $\Phi_A \approx$ 0.2. The highest polar order (Z≈1) is measured at ROI of $\approx$ 200 $\mu\mathrm{m}^2$, while as the ROI increases the Z decreases, hinting at local polar order of the system.

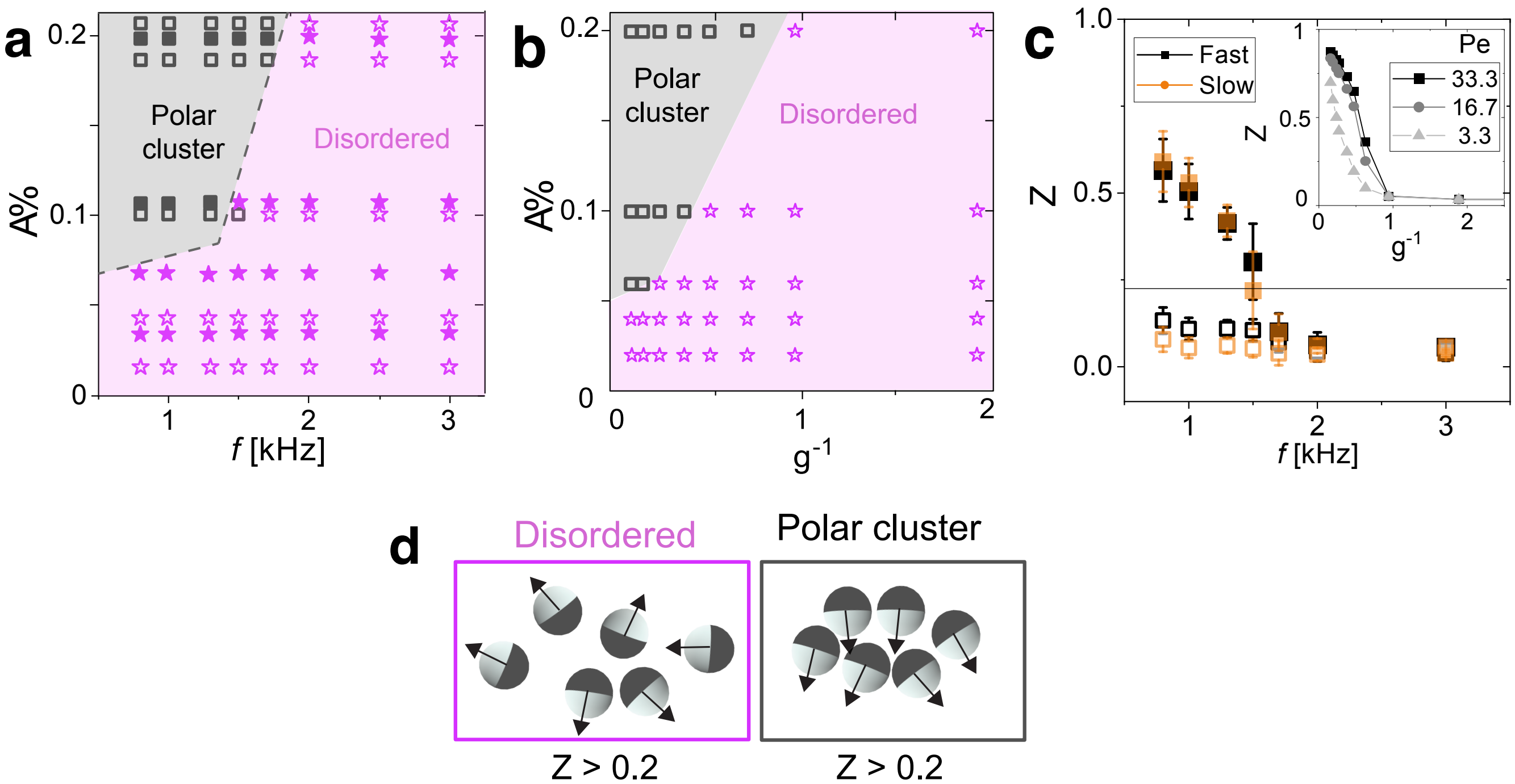

Fig. S4. **a,** Experimental phase diagram of the collective behaviour of fast $\mathrm{SiO_2}$-Pd (open symbols) and slow $\mathrm{SiO_2}$-Au (solid symbols) particles as a function of area fraction $\mathrm{A\%}$ and applied frequency f (kHz) at a fixed voltage of 8 $\mathrm{V_{pp}}$. **b,** Phase diagram obtained from numerical simulations at $\mathrm{Pe} = 13.3$, varying $\mathrm{A\%}$ and alignment $\mathrm{g^{-1}}$. **c,** Global polarization Z as a function of frequency for A%≈ 0.01 (open), and A%≈ 0.2 (solid) for slow $\mathrm{Pe^{exp}_{slow}} \approx 60$ and fast $\mathrm{Pe^{exp}_{fast}} \approx 130$ particles (orange and black symbols, respectively). Inset represents the results from numerical simulations at matching values of $\mathrm{g^{-1}}$ at $\mathrm{A\%} \approx 0.2$ for $\mathrm{Pe^{sim}} = 3.3$, $\mathrm{Pe^{sim}} = 16.7$ and $\mathrm{Pe^{sim}} = 33.3$ (from black to light grey). **c,** Schemes depicting the various states for disordered (magenta), polar cluster (gray) and single alignment (blue) events, corresponding to each phase of the diagram.

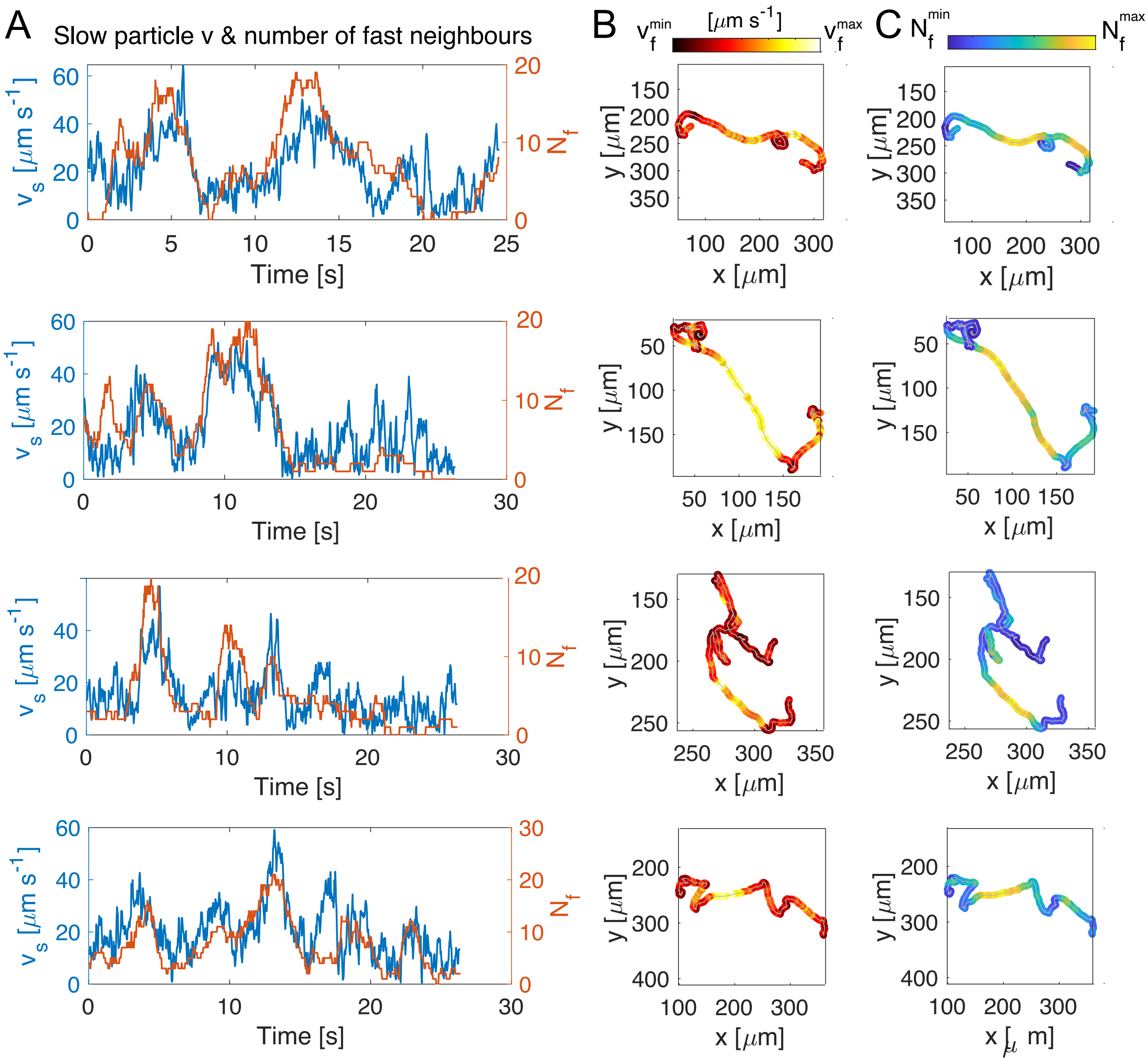

Fig. S5. Slow particle dynamics with respect to fastneighbours. **A** Velocity of a slow particle $\mathrm{v_s}$ with dt = 0.1s (blue) and number of slow neighbours $\mathrm{N_f}$ (orange) in a radius of 4 particles diameters as a function of time for 4 different slow particle trajectories. Corresponding particle trajectories in space depicting **B** their instantaneous velocity evolution and **C** the number of fast neighbours.

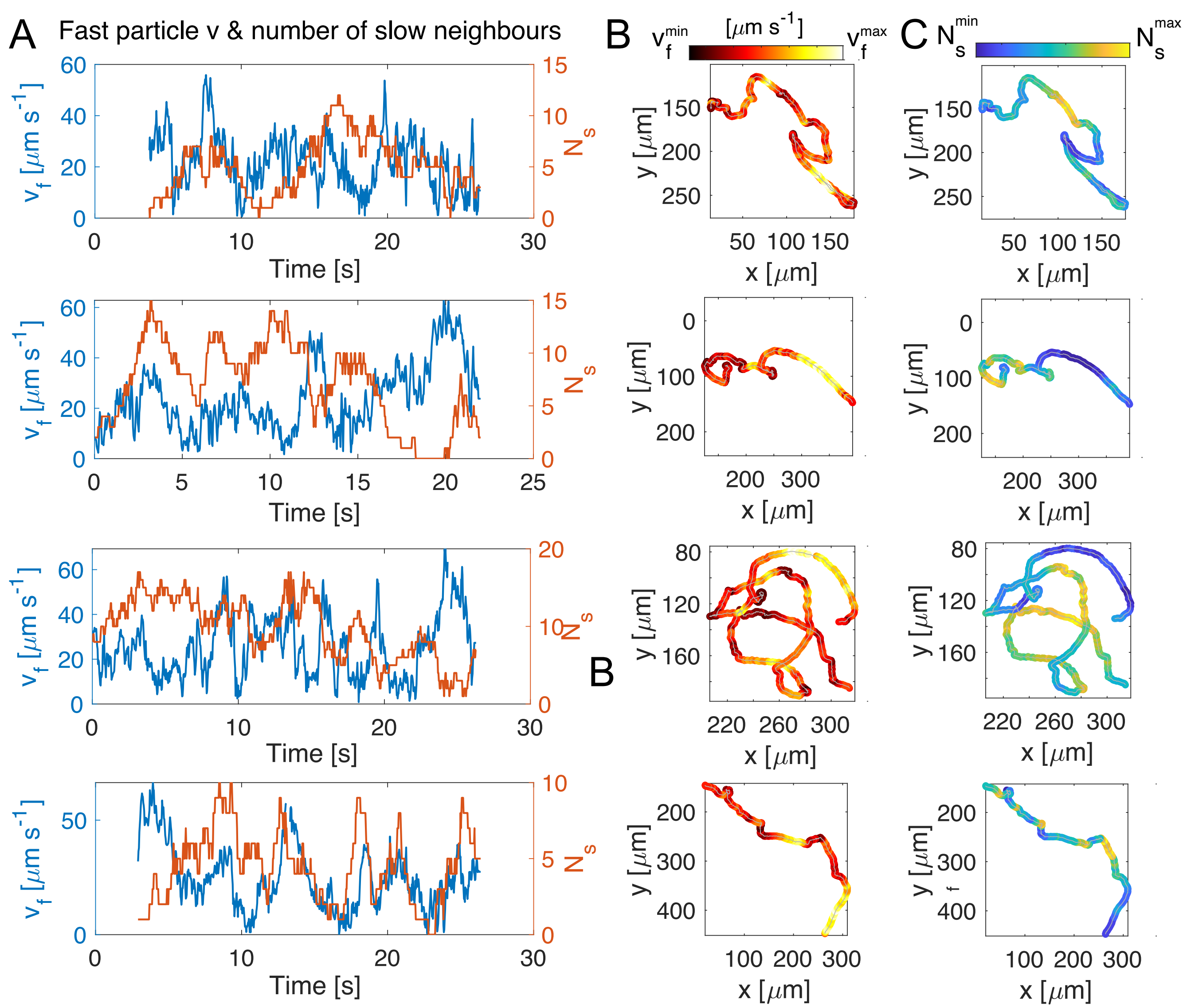

Fig. S6. Fast particle dynamics with respect to slow neighbours. **A** Velocity of a fast particle v_f with dt = 0.1s (blue) and number of slow neighbours N_s (orange) in a radius of 4 particles diameters as a function of time for 4 different slow particle trajectories. Corresponding particle trajectories in space depicting **B** their instantaneous velocity evolution and **C** the number of slow neighbours.

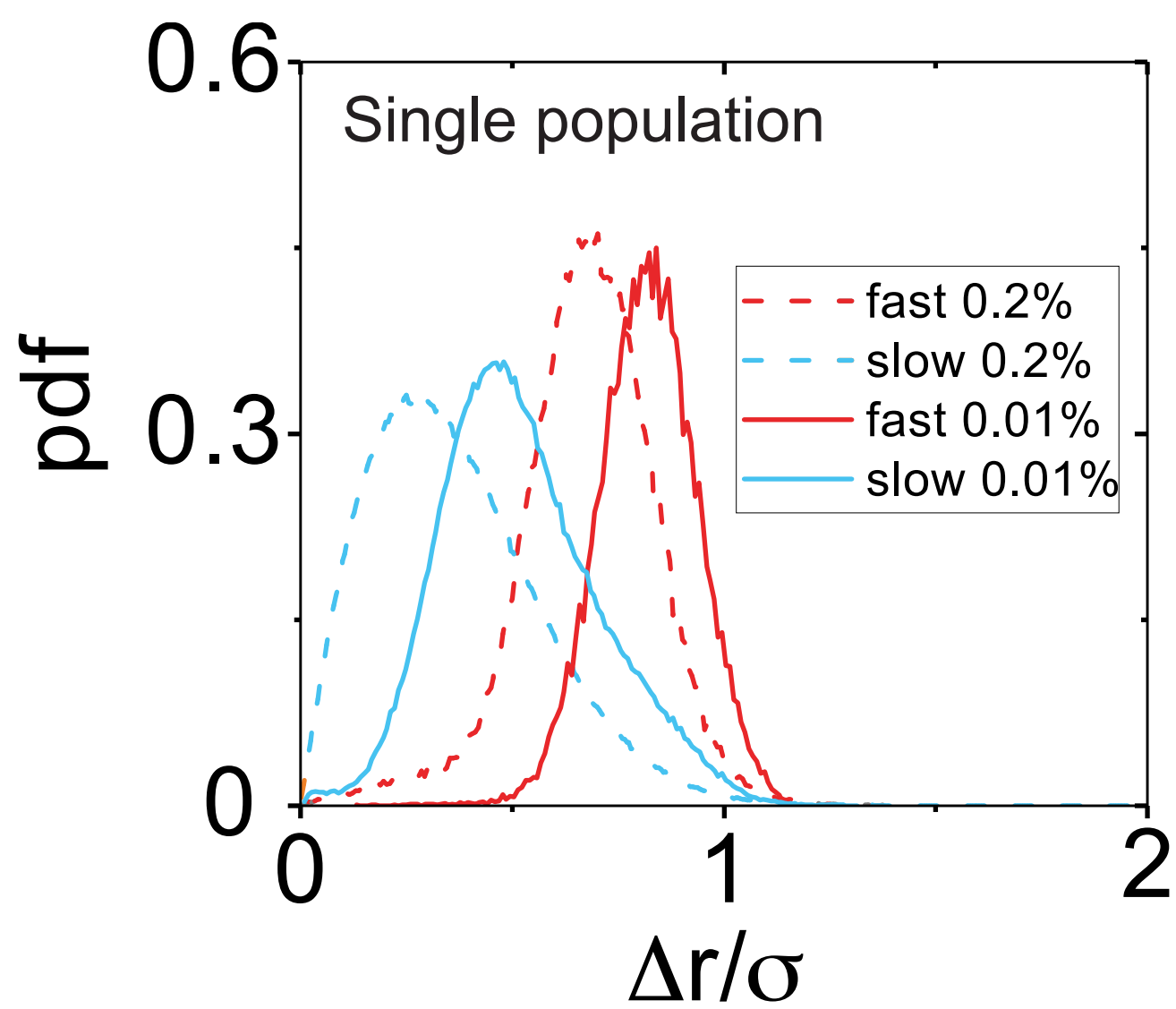

Fig. S7. a Experimental probability distribution of displacements for monodisperse samples of fast (red) and slow (cyan) Janus particles at diluted (0.01 Φ_A, solid line) and dense (0.2 Φ_A, dashed line) regimes. An effective decrease in the particle displacements due to crowding is observed as expected from the dilute to the dense regimes.

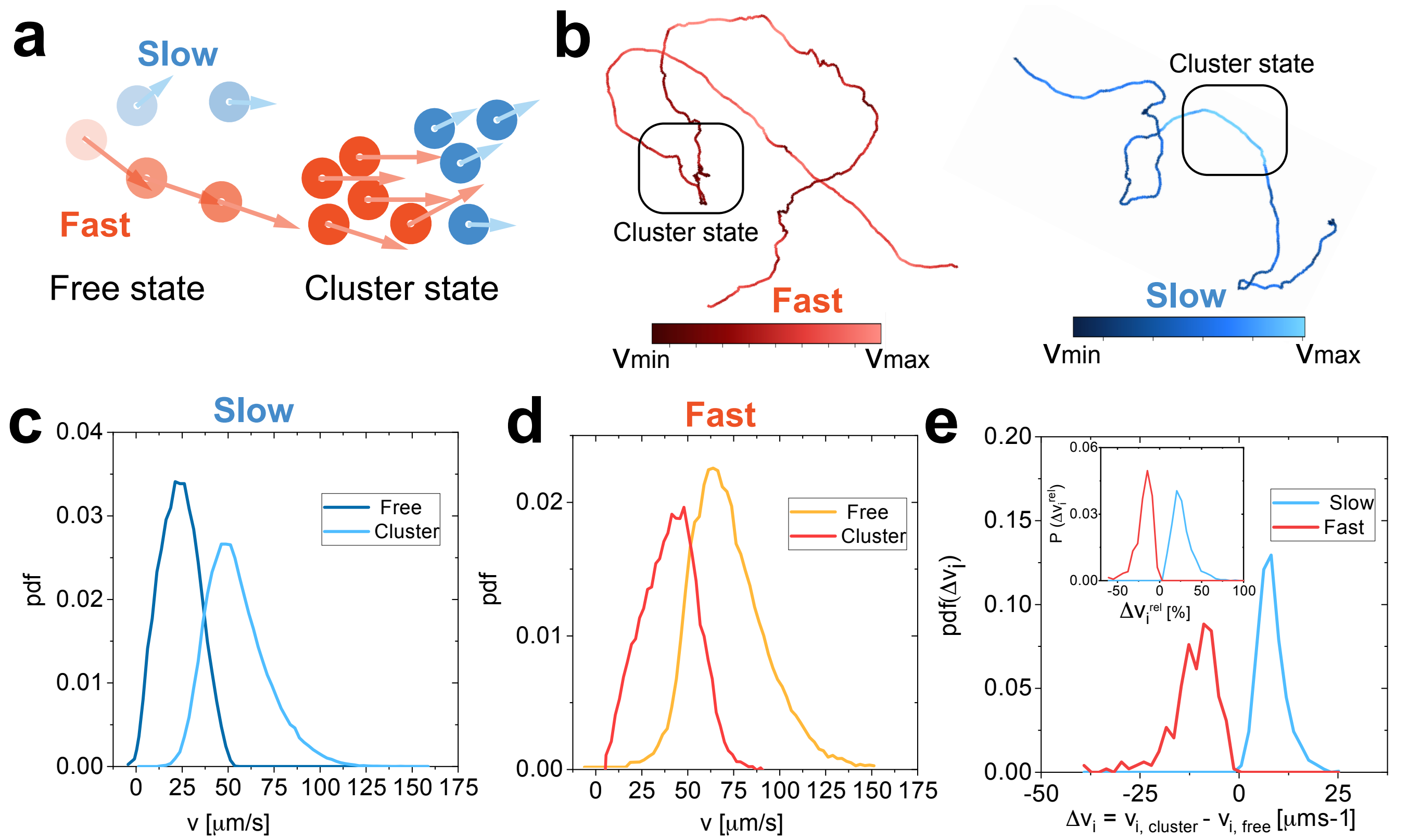

Fig. S8. **a**, Schematic representation of the free and cluster states for slow and fast particles. Within clusters, fast particles push and entrain slow particles, increasing the displacement of the slow population while reducing that of the fast population. **b**, Representative fast-particle (red) and slow-particle (blue) trajectories, color-coded by the local displacement magnitude. Black boxes indicate trajectory segments assigned to the cluster state. **c**, Probability density distribution of the in-plane displacement, d_{xy} at δt = 0.03 s, for slow particles in the free and cluster states. **d**, Corresponding displacement distributions for fast particles. Slow particles shift toward larger displacements in the cluster state, whereas fast particles shift toward smaller displacements, revealing opposite dynamical responses during collective motion. **e**, Probability density distributions of the particle-wise velocity difference between cluster and free states, $\Delta \mathrm{v_i} = \mathrm{v_{i,cluster}} - \mathrm{v_{i,free}}$, calculated for individual treajectories for their free and cluster states. Positive values indicate an increase in velocity in the cluster state, whereas negative values indicate a decrease. Slow particles exhibit a median velocity increase of $+7.8\ \mu m, s^{-1}$, while fast particles exhibit a median decrease of $-10.3\ \mu m, s^{-1}$. The inset shows the corresponding particle-wise relative velocity change, $\Delta \mathrm{v_i^{rel}} = 100(\mathrm{v_{i,cluster}} - \mathrm{v_{i,free}})/\mathrm{v_{i,free}}$, for which the median changes are $+24.4\%$ and -16.1% for slow and fast particles, respectively.

Supplementary Movies

Movie S1. Experimental movie of a semi-dense phase ($\Phi_A \approx 0.15$) of fast particles transitioning from elastic collisions (2 kHz, $g^{-1} > 1$) to polar alignment (1 kHz, $g^{-1} < 1$) at a fixed voltage 8 V_{pp}.

Movie S2. Numerical movie depicting the effect of Pe at a fixed g^{-1} =0.2. The colours indicate the individual particle velocity orientation.

Movie S3. Experimental binary mixtures movies at $\Phi_A \approx 0.2$ at 1 kHz and 8V_{pp}. The movie on the left depicts the population coexistence with the slow (blue) and fast (red) ($x_F \approx 0.47$). The arrows indicate the magnitude of the velocity. The movie on the right is the corresponding representation of the velocity vector magnitude and orientations in different colours.

Movie S4. Numerical binary mixtures movies at $\Phi_A \approx 0.2$ at g^{-1} =0.2, as analogous to Movie S2. The movie on the left depicts the population coexistence with the slow (blue) and fast (red) with Pe 3.3 and 13.3, respectively ($x_F \approx 0.5$). The movie on the right is the corresponding representation, where the colours represent the particle velocity orientation

Movie S5. Movie of an event of a single slow particle being pushed by a fast cluster. The coloured trajectory depicts the instantaneous velocity of the slow particle (blue) at 1 kHz and 8V_{pp}.

Movie S6. Numerical experiments for binary mixtures at $\Phi_A = 0.2$ for fixed alignment g^{-1} =0.2 at varying Pe.
